# Syndromic Multiplex Polymerase Chain Reaction: The Impact on Microbial Yield in Nonventilator Hospital-Acquired Pneumonia

**DOI:** 10.1093/ofid/ofag272

**Published:** 2026-05-05

**Authors:** Jon Anders Feet, Karl Erik Müller, Øyvind Kommedal, Kathrine Brun, Elling Ulvestad, Harleen M S Grewal, Lars Heggelund

**Affiliations:** Department of Clinical Science, Bergen Integrated Diagnostic Stewardship Cluster, Faculty of Medicine, University of Bergen, Norway; Department of Medicine, Drammen Hospital, Vestre Viken Hospital Trust, Drammen, Norway; Department of Clinical Science, Bergen Integrated Diagnostic Stewardship Cluster, Faculty of Medicine, University of Bergen, Norway; Department of Medicine, Drammen Hospital, Vestre Viken Hospital Trust, Drammen, Norway; Department of Clinical Science, Bergen Integrated Diagnostic Stewardship Cluster, Faculty of Medicine, University of Bergen, Norway; Department of Microbiology, Haukeland University Hospital, Bergen, Norway; Department of Clinical Science, Bergen Integrated Diagnostic Stewardship Cluster, Faculty of Medicine, University of Bergen, Norway; Department of Microbiology, Haukeland University Hospital, Bergen, Norway; Department of Clinical Science, Bergen Integrated Diagnostic Stewardship Cluster, Faculty of Medicine, University of Bergen, Norway; Department of Microbiology, Haukeland University Hospital, Bergen, Norway; Department of Clinical Science, Bergen Integrated Diagnostic Stewardship Cluster, Faculty of Medicine, University of Bergen, Norway; Department of Clinical Science, Bergen Integrated Diagnostic Stewardship Cluster, Faculty of Medicine, University of Bergen, Norway; Department of Medicine, Drammen Hospital, Vestre Viken Hospital Trust, Drammen, Norway

**Keywords:** antimicrobial resistance, antimicrobial therapy, clinical course, multiplex polymerase chain reaction, nonventilator hospital-acquired pneumonia

## Abstract

**Background:**

Nonventilator hospital-acquired pneumonia (NV-HAP) is a leading cause of hospital-acquired infection, associated with substantial morbidity and mortality. Conventional bacterial cultures from lower respiratory tract specimens, as recommended by current guidelines, have limited sensitivity and frequently fail to identify causative pathogens. This study evaluated a rapid, culture-independent molecular diagnostic approach versus standard methods for identifying microbial etiology, assessing antimicrobial prescribing, and determining NV-HAP incidence in a medium-sized Norwegian hospital. We hypothesized that multiplex polymerase chain reaction (mPCR) would shorten time to diagnosis and increase microbial yield.

**Method:**

A screening process twice daily was established to prospectively identify NV-HAP patients eligible for inclusion in a single hospital from 11 June 2021 to 26 November 2024. Lower respiratory tract samples were analyzed using a syndromic multiplex PCR assay and compared with conventional culture methods.

**Results:**

Eighty-six patients were included, with bacterial pathogens detected in 51.2% of the samples analyzed by mPCR and in 23.5% of cultured samples (*P* < .01). Median time to microbiological results were 2.5 (2–10) hours for mPCR and 45.8 (26.9–50.3) hours for bacterial culture (*P* < .01). Overall agreement was fair to moderate (κ = 0.39), with an observed agreement of 69%. Antibiotic therapy was modified based on the mPCR in 13 patients (15.1%) and due to culture in 9 patients (10.5%).

**Conclusions:**

The use of syndromic mPCR provided faster results and a higher detection rate for microbial etiology compared to traditional culture methods.

## Graphical Abstract

**Graphical Abstract:**
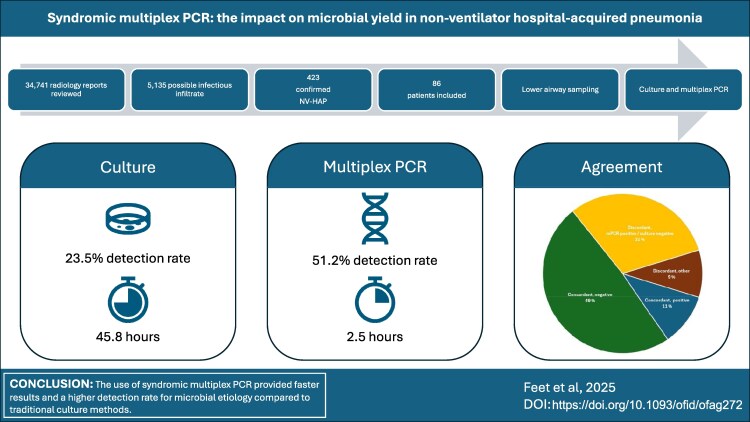

## Teaser

Multiplex polymerase chain reaction (PCR) identified bacteria in 51% of samples versus 24% by culture (*P* < .01). Multiplex PCR provided significantly faster results (2.5 hours vs 45.8 hours; *P* < .01). Overall agreement was fair to moderate (κ = 0.39), with an observed agreement of 69%.

Hospital-acquired pneumonia (HAP) is often divided into nonventilator hospital-acquired pneumonia (NV-HAP) and ventilator-associated pneumonia (VAP), with most research performed on the latter [1]. The European Centre for Disease Prevention and Control estimates that NV-HAP accounts for 64% of HAPs and 13.7% of all hospital-acquired infections [2]. Current literature estimates an NV-HAP incidence of 1.3–5.9 per 1000 patient-days and 4.9–21.2 per 1000 hospital admissions [3–7]. Nonventilator hospital-acquired pneumonia extends hospital stay by 4–16 days [8, 9], and the reported in-hospital mortality rate is 13%–30% [4, 5, 7–11]. The most common pathogens identified by traditional methods are Gram-negative bacilli and *Staphylococcus aureus* [12]. For microbial diagnosis, national and international guidelines recommend standard bacterial culture, which has a low detection rate [13]. Empirical treatment of HAP commonly involves the use of broad-spectrum antibiotics to ensure early coverage of potential multidrug-resistant pathogens. However, guidelines recommend avoiding superfluous treatment that may lead to adverse drug effects, *Clostridioides difficile* infections, antibiotic resistance, and increased cost [12].

Results from conventional bacterial cultures are usually available after 36–72 hours [14]. Commercial real-time multiplex polymerase chain reaction (mPCR) tests represent a novel alternative that can deliver results in less than 2 hours and offer higher detection rates than traditional culture methods. Potentially, this would enable earlier customization and possibly de-escalation of antimicrobial therapy; however, such tests have not yet been incorporated into clinical guidelines [13].

Internationally, there are few studies on NV-HAP outside of the intensive care environment. Clinical guidelines for NV-HAP are supported largely by data from VAP studies, which may not be representative for NV-HAP [15].

Increased knowledge of microbial agents, resistance patterns, and management of NV-HAP is urgently needed. The use of syndromic multiplex PCR in VAP and NV-HAP has only recently emerged as a suitable alternative to standard methods [16].

The aim of this study was to evaluate the utility of a comprehensive, culture-independent, rapid molecular diagnostic approach compared with a standard diagnostic approach for determining microbial etiology, reviewing antimicrobial prescribing, and assessing the incidence and etiology of NV-HAP in a medium-sized Norwegian hospital. Our primary hypothesis was that the use of multiplex PCR would significantly reduce the time to microbiological diagnosis. Secondarily, we hypothesized that a more comprehensive microbial diagnostic strategy would increase microbial yield. Finally, we aimed to determine the incidence of NV-HAP in a general hospital setting and evaluate antimicrobial prescribing practices in relation to national guidelines.

## METHODS

### Study Design and Participants

We performed a single-center, prospective study of patients hospitalized at Drammen Hospital, Vestre Viken Hospital Trust, a medium-sized Norwegian hospital serving a local population of approximately 185 000 residents and functioning as a referral hospital for nearly 500 000 people. It provides comprehensive medical, oncological, and surgical services across all major specialties except thoracic surgery and neurosurgery and does not serve as a transplant center. The trial, titled HVAPNOR, was registered at ClinicalTrials.gov (NCT04381247). Patients were included between 11 June 2021 and 26 November 2024. Over the course of the study period, a total of 92 551 hospital admissions and 279 327 patient-days were recorded among 59 657 unique individuals. The definition of NV-HAP was extrapolated from the Infectious Diseases Association of America/American Thoracic Society (IDSA/ATS) 2016 guidelines for the treatment of HAP [12].

### Data Source, Method for Selection, and Eligibility Criteria

A search in the hospital radiology software was set up to include all reports of X-rays and computed tomography (CT) scans of the thorax performed on adult inpatients aged 18 years or older. All radiology reports were signed by a specialist in radiology. The reports extracted from the search were reviewed twice daily on weekdays by a study investigator to ascertain if the report described radiological findings of possible infectious origin. For patients with a possible infectious infiltrate, investigators proceeded to review electronic hospital charts. If the patient was considered eligible for inclusion, the investigator would proceed to discuss inclusion with the treating physician before obtaining consent and completing the inclusion process.

Inclusion criteria were (1) clinical suspicion of pneumonia with a new or progressing radiographic infiltrate in a patient admitted for at least 48 hours or readmitted less than 48 hours after discharge from hospital and (2) at least 2 of the following clinical features: (a) temperature ≥ 38.0°C or <36.0°C, (b) white blood cell (WBC) count > 11.0 × 10^9^ or <3.5 × 10^9^ cells/L, (c) purulent airway secretions, or (d) reduced oxygenation. Reduced oxygenation was defined as a peripheral oxygen saturation (SpO_2_) decline of ≥5% with an unchanged fraction of inspired oxygen (FiO_2_) or any reduction in the ratio of arterial oxygen partial pressure (PaO_2_) to FiO_2_ (P/F ratio).

We excluded cases where (1) the patient received treatment for community-acquired pneumonia (CAP) with no suspicion of secondary infection, (2) the treating clinician did not consider pneumonia to be a likely diagnosis, (3) the patient could not produce a lower airway sample, (4) the patient was treated with mechanical ventilation for more than 48 hours, (5) the patient had refractory septic shock, (6) patients treated with antibiotics for NV-HAP > 72 hours, (7) patients with a life expectancy of <1 week, and (8) patients with a Glasgow coma scale score of 3.

### Data Collection and Management

We reviewed electronic patient charts and recorded demographic variables and clinical scores at the time of inclusion, including sex, age, comorbidities, admission types, Charlson Comorbidity Index (CCI) score, and Clinical Frailty Scale (CFS) score.

Clinical parameters were systematically recorded from electronic patient charts on days 0 and 2, and after treatment, including the Sequential Organ Failure Assessment (SOFA) score, Simplified Acute Physiology Score 2 (SAPS-2), National Early Warning Score 2 (NEWS2), length of hospital stay, mortality outcomes, microbiological findings, and data on antimicrobial therapy administered throughout the study period. The following microbiological samples were collected: blood cultures, lower airway samples (induced sputum, endotracheal aspirate, or bronchoalveolar lavage), and urine for antigen testing. Additionally, blood and microbiological samples were harvested for analysis and biobanking.

### Microbiological Analysis

Standard diagnostics encompassed bacterial cultures and in-house PCR panels for atypical bacterial and viral agents. The samples were processed according to the procedures and guidelines of the local microbiological department. Identification of bacterial isolates was made by matrix-assisted laser desorption/ionization time-of-flight mass spectrometry (MALDI-TOF MS) with the MALDI Biotyper system (Bruker Daltonics, Bremen, Germany), and antimicrobial resistance testing was performed on all isolates deemed clinically relevant.

In parallel, all lower respiratory tract samples were analyzed using the BioFire® FilmArray® Pneumonia Plus Panel (BioFire Diagnostics, Salt Lake City, UT, USA). This syndromic multiplex PCR test integrates nucleic acid extraction, reverse transcription, and nested multiplex PCR amplification to detect 18 bacterial pathogens, 9 viral targets, and 7 antimicrobial resistance genes (Supplementary Table 1), providing rapid, random-access, and comprehensive pathogen identification. The panel produces semiquantitative results for potentially pathogenic bacteria in the range from 10^4^ to >10^7^ copies/mL.

### Antibiotic Treatment Guidelines

Norwegian treatment guidelines differentiate recommended antibiotic regimens for NV-HAP based on the risk of a complicated disease course. Risk factors include (1) length of hospital stay, (2) clinical condition, (3) comorbidities, (4) patient age, and (5) the risk of resistant pathogens. For low-risk patients, the recommended regimen is benzylpenicillin (1.2 g 4 times daily) combined with gentamicin (6 mg/kg once daily), with cefotaxime (1 g 3 times daily) as an alternative. For high-risk patients, piperacillin/tazobactam (4 g/0.5 g 3 times daily) is recommended, with meropenem (1 g 3 times daily) as an alternative.

### Statistical Analysis

Statistical analyses were performed using StataNow/SE 18.5. Results are expressed as counts and proportions for categorical variables. Continuous variables are expressed as median with interquartile range (IQR). Incidence data are expressed as NV-HAP cases per 1000 admissions and per 1000 patient-days. Statistical significance was assessed using the Wilcoxon signed-rank test when assessing time from airway sampling to microbiological result. McNemar's test was used when assessing differences in paired detection rates between multiplex PCR and culture. Overall agreement was assessed at the sample level using Cohen's к for detection of any pathogen, while organism-specific к values were calculated separately for each pathogen included on both panels. Multivariable logistic regression was performed to assess factors hypothesized to be associated with test performance.

## RESULTS

### Study Population

**Figure 1.**
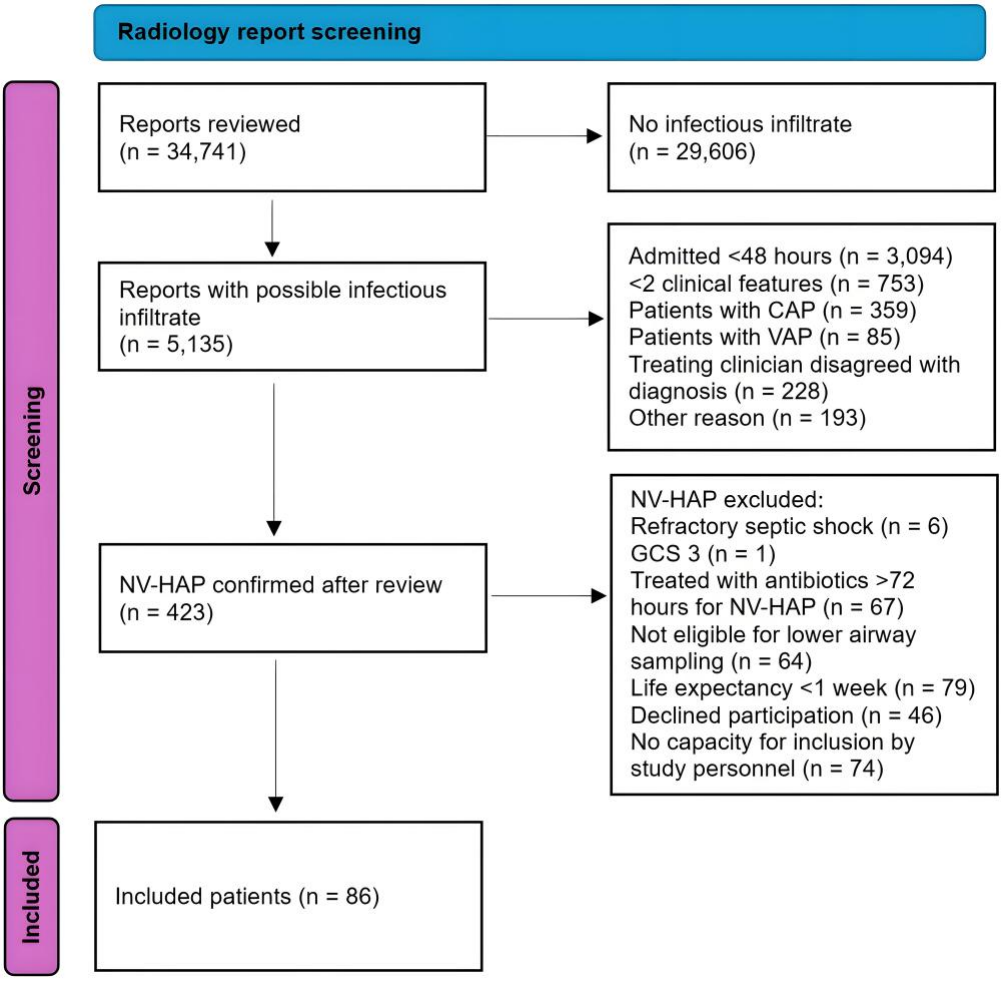
Radiology report screening process. Overview of the screening and inclusion process.

A total of 34 741 radiology reports were evaluated during the study period, from which 86 out of 423 patients deemed likely to have NV-HAP were eligible for inclusion (Figure 1). The incidences of NV-HAP were 4.6 per 1000 admissions and 1.5 per 1000 patient-days. Demographics, comorbidities, admission type, and prevalence of the different inclusion criteria are detailed in Table 1. Chest X-rays (CXR) were performed in 74 cases (86%), and chest CT was performed in 19 cases (22.1%), 9 of whom also underwent CXR. The pulmonary examination was abnormal in 74.6% of patients. Active smokers (15.5%) and previous smokers (46.4%) had 22.4 (IQR 6.1–37.9) pack-years. Twenty-two (25.6%) patients had leukopenia at the time of inclusion. Most patients lived at home with no assistance (62.8%) at the time of admission, and no patient resided in a nursing home. However, at the time of discharge, only 21.9% went home without assistance, and 39% were discharged to nursing homes.

**Table 1.** Demographics, Comorbidities, Admission Types, Prevalence of Inclusion Criteria, and Place of Residence on Admission and at Discharge

Demographics	
Male sex, no. (%)	55 (64%)
Age, y	75 (65–81)
Clinical Frailty Scale score	3 (3–5)
Comorbidities	
Charlson Comorbidity Index score	5 (4–7)
Obstructive pulmonary disease, no. (%)	24 (27.9%)
Coronary artery disease, no. (%)	22 (25.6%)
Heart failure, no. (%)	17 (19.8%)
Chronic kidney disease, no. (%)	15 (17.4%)
Type 2 diabetes, no. (%)	18 (20.9%)
Solid organ cancer, no. (%)	14 (16.3%)
Leukemia or lymphoma, no. (%)	10 (11.6%)
Admission type	
Medical, no. (%)	56 (65.1%)
Surgery orthopedic, no. (%)	15 (17.4%)
Surgery other, no. (%)	15 (17.4%)
Inclusion criteria	
Temperature, no. (%)	55 (64%)
Leukocytes, no. (%)	64 (74.4%)
Purulent secretions, no. (%)	73 (84.9%)
Reduced oxygenation, no. (%)	60 (69.8%)
Place of residence on admission	
Own home, no assistance, no. (%)	54 (62.8%)
Own home, assistance from relative(s) or home nursing, no. (%)	30 (34.9%)
Residential care, no. (%)	2 (2.3%)
Nursing home, no. (%)	0 (0%)
Place of residence at discharge	
Own home, no assistance, no. (%)	14 (21.9%)
Own home, assistance from relative(s) or home nursing, no. (%)	19 (29.7%)
Residential care, no. (%)	2 (3.1%)
Nursing home, no. (%)	25 (39.0%)
Unknown, discharge to other hospital, no. (%)	4 (6.3%)

Data are presented as number (percent) or median (IQR lower–IQR upper).

N = 86, except for discharge, where N = 64 due to in-hospital deaths.

At inclusion, the SAPS-2 was 32 (IQR 26–40), the SOFA score was 3 (IQR 2–4), and the NEWS2 was 7 (IQR 5–10). Clinical outcomes are detailed in Table 2.

**Table 2.** Clinical Outcomes

Clinical Outcomes		N
Time to NV-HAP, d	8 (5–14)	86
Length of hospital stay, d	17.1 (11.4–26.2)	86
ICU^a^ admission, no. (%)	35 (40.7%)	86
Length of ICU stay^b^, d	3.9 (2.4–10)	35
Invasive mechanical ventilation, no. (%)	14 (16.3%)	86
Length of mechanical ventilation^c^, d	8.1 (4.1–21.2)	14
In-hospital mortality, no. (%)	14 (16.3%)	86
30-day mortality accumulated, no. (%)	23 (26.7%)	86
90-day mortality accumulated, no. (%)	25 (29.1%)	86

Data are presented as number (percent) or median (IQR lower–IQR upper).

^a^ICU, intensive care unit.

^b^Among patients requiring treatment in the ICU.

^c^Among patients requiring invasive mechanical ventilation.

### Microbiological Sampling and Time to Diagnosis

Induced sputum was collected from 73 (84.9%), endotracheal aspirate (ETA) from 8 (9.3%), and bronchoalveolar lavage (BAL) from 5 (5.8%) patients. Endotracheal aspirate was performed by trained personnel on awake patients who did not manage to provide induced sputum. Bronchoalveolar lavage was performed if desired by the treating clinician. Of the 86 samples collected, one sample went missing, and one sample was not examined with the syndromic multiplex PCR. The median time from airway sampling to microbiological results was 2.5 (IQR 2–10) hours for syndromic multiplex PCR and 45.8 (IQR 26.9–50.3) hours for bacterial culture (*P* < .01).

### Microbiological Findings

**Figure 2.**
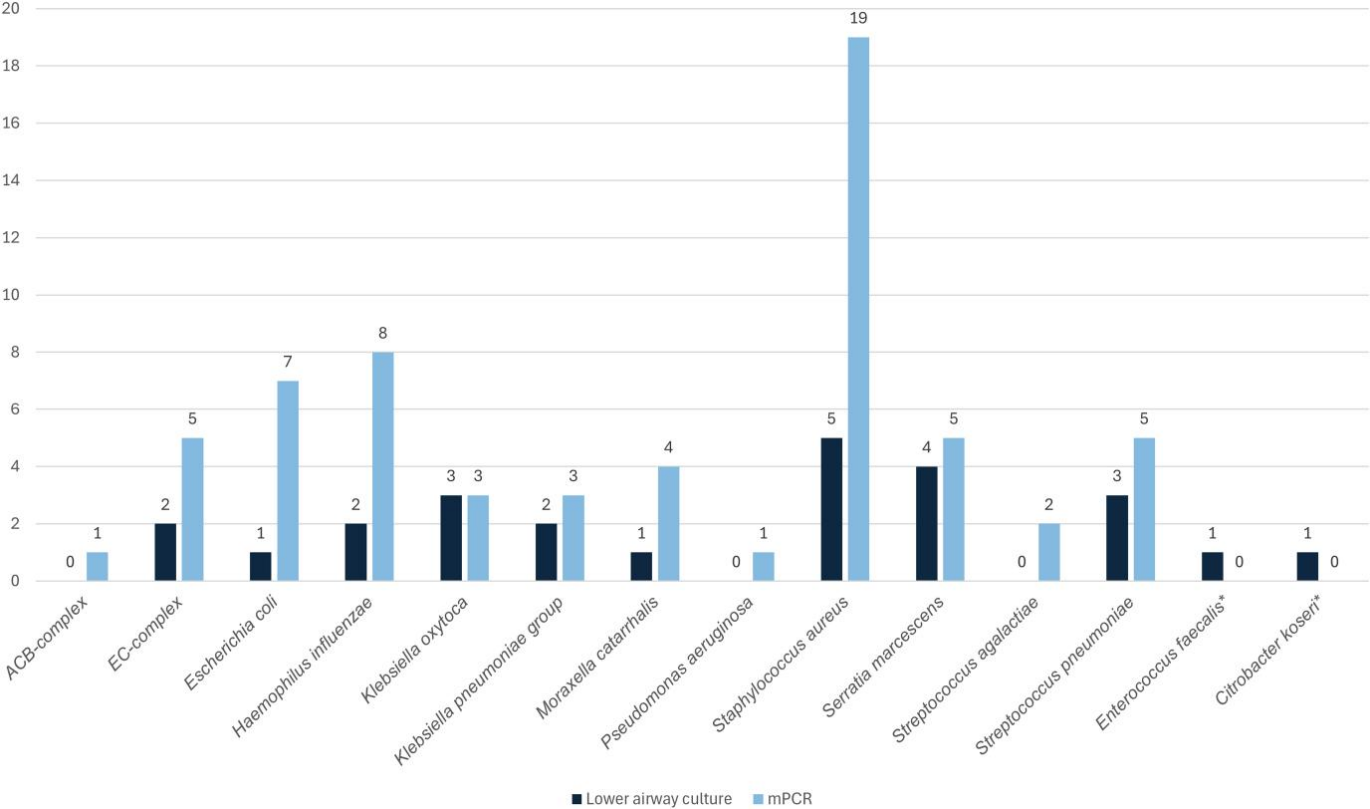
Bacterial identifications. Figure detailing bacterial identifications for mPCR and lower airway culture. *Enterococcus faecalis and *Citrobacter koseri* are not included in the mPCR panel. ACB complex, *Acinetobacter calcoaceticus–baumannii* complex; EC complex, *Enterobacter cloacae* complex; mPCR, multiplex polymerase chain reaction.

Microbial detections were made in 52.4% of samples. The syndromic multiplex PCR detected potential pathogens in 51.2% of samples, while culture detected potential pathogens in 23.5% of samples (*P* < .01). The most frequently detected bacteria were Gram-negative rods and *S. aureus*. Bacterial identifications are detailed in Figure 2, with complete details including semiquantitative results in Supplementary Table 2. Syndromic multiplex PCR identified multiple organisms within individual samples in 16 cases: 2 organisms in 13 samples, 3 in 2 samples, and 4 in 1 sample. In contrast, culture yielded multiple identifications in only 3 cases: 2 organisms in 1 sample and 3 organisms in 2 samples. Overall agreement between multiplex PCR and culture for detection of any bacterial pathogen was fair to moderate (κ = 0.39), with an observed agreement of 69%. Pathogen-specific agreement between FilmArray and culture varied across organisms. Substantial agreement was observed for *Klebsiella pneumoniae* group (κ = 0.79), *Streptococcus pneumoniae* (κ = 0.74), *Klebsiella oxytoca* (κ = 0.65), and *Serratia marcescens* (κ = 0.65), all with observed agreement exceeding 96%. Moderate to fair agreement was observed for *Haemophilus influenzae* (κ = 0.38), *Moraxella catarrhalis* (κ = 0.39), *Escherichia coli* (κ = 0.23), *Enterobacter cloacae* complex (κ = 0.26), and *S. aureus* (κ = 0.33). Agreement and Cohen's kappa calculations are detailed in Supplementary Table 4.

**Figure 3.**
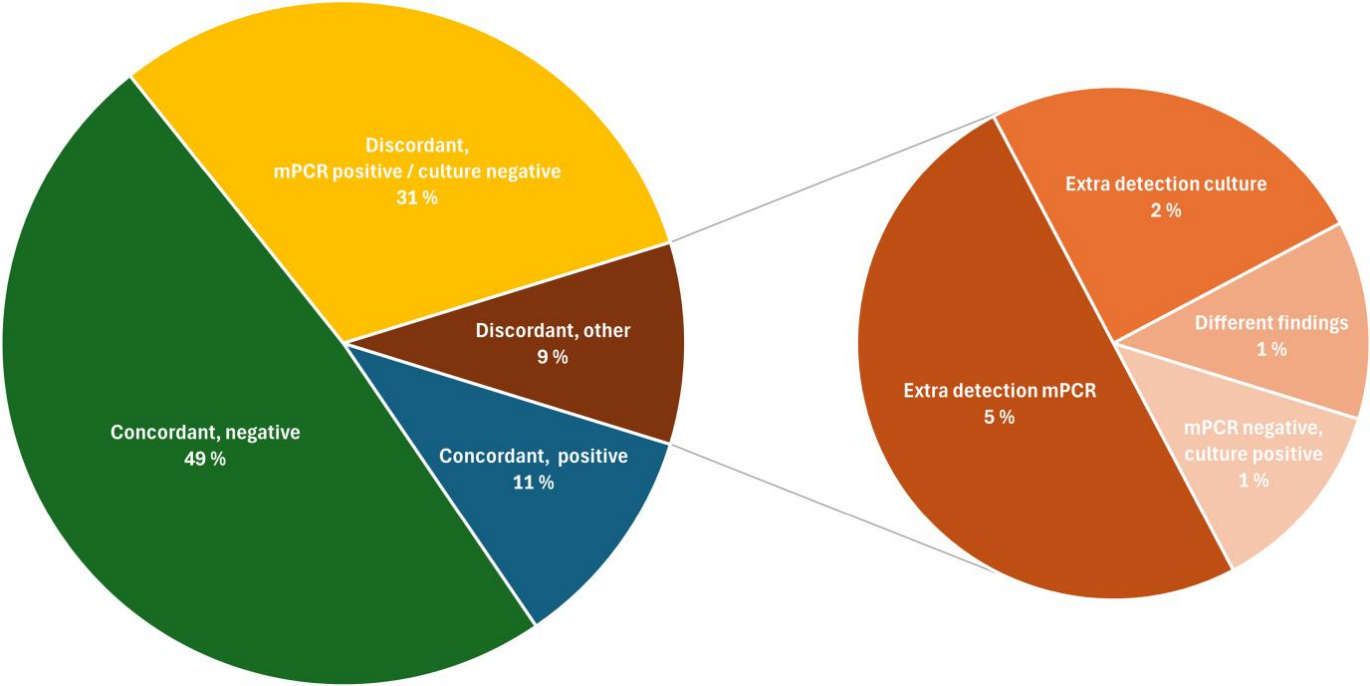
Agreement between syndromic multiplex PCR and culture. Figure displaying details on concordance and discordance between multiplex polymerase chain reaction (mPCR) and airway culture.

Concordance between syndromic multiplex PCR and culture was observed in 60% of the samples (Figure 3). The main reason for discordance was a positive syndromic multiplex PCR and negative culture (Figure 3).

The syndromic multiplex PCR detected resistance genes for *mec*A, *mec*C, and SCC*mec* right-extremity junction (MREJ) in 3 samples, but no resistance genes for extended-spectrum beta-lactamase or carbapenems were detected. Antibiotic susceptibility testing for empirical treatment regiments is detailed in Supplementary Table 3.

Viruses were identified in 7 samples with the syndromic multiplex PCR and 5 samples with the in-house PCR. The syndromic multiplex PCR detected 5 influenza A, 1 human rhinovirus/enterovirus, and 1 parainfluenza virus. The in-house PCR identified 4 influenza A and 1 parainfluenza virus, resulting in concordance for 97.6% of samples. In addition, the in-house PCR detected severe acute respiratory syndrome coronavirus 2 (SARS-CoV-2) in 13 (15.3%) samples.

In 47% of airway cultures, growth of *Candida* spp. was observed, usually interpreted as colonization. In multivariable logistic regression analyses adjusting for age, comorbidity burden, and ongoing antibiotic therapy at the time of sampling, the presence of yeast in airway cultures was inversely associated with bacterial positivity (odds ratio (OR), 0.31; 95% CI, .10–1.00; *P* = .049). In contrast, no significant association was observed between yeast presence and multiplex PCR positivity (OR, 0.50; 95% CI, .20–1.26; *P* = .14). Age, CCI score, and ongoing antibiotic therapy at the time of sampling were not independently associated with either outcome.

### Antibiotic Prescription

Within the 14-day period prior to inclusion, 62.3% of patients had completed an antibiotic course. At the time of inclusion, 75.6% had ongoing antibiotic therapy, of which 73.8% had been initiated for suspected NV-HAP. Initial antibiotic treatment adhered to national empirical guidelines in 86.7% of patients. Antibiotic therapy was modified based on the syndromic multiplex PCR results in 13 patients (15.1%) and based on culture findings in 9 patients (10.5%). Among patients whose therapy was modified following multiplex PCR, escalation occurred in 4 cases, de-escalation in 6 cases, and modification within the same antibiotic spectrum class in 3 cases. In contrast, culture-guided modifications resulted in escalation in 1 case, de-escalation in 7 cases, and modification within the same spectrum class in 1 case. The median duration of antibiotic treatment was 8 (5–12) days.

Six patients received antiviral treatment, including all 5 patients with influenza A and 1 with SARS-CoV-2.

## DISCUSSION

Defining a case of NV-HAP is difficult and comprises an inherent uncertainty. Surveillance data and incidence numbers are therefore troublesome. Surveillance is further complicated by the fact that NV-HAP does not have a specific International Classification of Diseases 10 (ICD10) diagnosis. In this study, radiology reports were screened twice daily over a 3-year period to obtain the most robust incidence data possible. The incidence of NV-HAP in our study was 4.6 per 1000 admissions and 1.5 per 1000 patient-days, which is in line with other publications [3–7].

We chose to define NV-HAP cases by the IDSA criteria, even though most patients solely underwent CXR, which has lower sensitivity compared to chest CT [17–20]. Nevertheless, CXR remains the primary radiological tool used in clinical practice for the evaluation of suspected NV-HAP, and we believe its use aligns with standard diagnostic procedures.

Bacterial detections were made in 52.4% of the patients. Although 75.6% of patients were receiving antibiotics for NV-HAP at the time of airway sampling, no association was observed between ongoing antibiotic therapy and failure to detect pathogens by either culture or multiplex PCR. In culture-based analyses, yeast colonization was inversely associated with bacterial positivity; however, this finding was characterized by a very wide CI and was derived from a small cohort. Accordingly, this result should be interpreted with caution.

A higher number of pathogen detections by the multiplex PCR panel compared to culture are in line with other studies [21, 22].

In this clinically defined cohort, agreement between multiplex PCR and conventional culture was overall fair to moderate, reflecting inherent differences between molecular and culture-based diagnostics. Substantial agreement was observed for several key respiratory pathogens, including *K. pneumoniae* group, *S. pneumoniae*, *K. oxytoca*, and *S. marcescens*, all with very high observed agreement. In contrast, moderate to fair κ values were seen for other organisms despite high observed agreement, a pattern consistent with the known dependence of κ on pathogen prevalence and imbalanced marginal distributions rather than true methodological discordance. For pathogens with no detections by 1 or both methods, κ could not be calculated because the absence of variability precludes estimation of chance-corrected agreement. Overall, these findings indicate good concordance between multiplex PCR and culture for several clinically important pathogens while underscoring the complementary nature of molecular diagnostics in respiratory infection assessment.

In our cohort, no resistance toward extended-spectrum beta-lactamase or carbapenems, either by syndromic multiplex PCR or by culture, was observed. Only 3 samples displayed *mec*A, *mec*C, and MREJ genes, and in one of these cases, targeted treatment with linezolid was administered due to this result.

Most of the patients received antibacterial therapy in accordance with the Norwegian national guidelines. More than 50% of patients received cefotaxime as initial treatment, followed by piperacillin/tazobactam and meropenem as the second and third most prescribed treatments, offering adequate Gram-positive and Gram-negative coverage for most identified pathogens considering the current state of antimicrobial resistance in Norway.

Rapid results from microbiological analyses are generally regarded as important in an antibiotic stewardship perspective [23]. In this study, the median time from sampling to a result available for clinicians was significantly shorter with syndromic multiplex PCR compared to bacterial culture, 2.5 versus 46 hours. In addition, a larger portion of the patients had microbiological detections. Both these observations are in line with another recently published study using this platform [16].

Antimicrobial therapy was modified in only 15.1% of patients based on syndromic multiplex PCR results and in 10.5% based on culture findings. The number of modifications was low, possibly in part reflecting a reluctancy among clinicians to de-escalate antibiotic treatment based on a new method with which they had little experience. The low number of modifications may also reflect the adequacy of the empirical Norwegian guidelines and the low level of antimicrobial resistance in the country.

In Norway, the Methicillin-resistant *Staphylococcus aureus* (MRSA) prevalence is low with 2942 cases reported in 2024, of which 1222 were reported as a clinical infection, and only 27 (2% of infections) represented blood stream infections [24]. Patients with prior healthcare exposure in high-MRSA-prevalence settings are routinely screened and isolated upon hospital admission. As a result, rapid PCR testing specifically to guide MRSA coverage in NV-HAP is not standard practice in Norway. Nevertheless, in high-MRSA-prevalence settings, rapid MRSA testing could be highly valuable, also in NV-HAP, as it would allow for earlier de-escalation or targeted escalation of empiric therapy, reducing unnecessary broad-spectrum antibiotic use.

The primary strengths of this study include its rigorous design, characterized by thorough and systematic screening of radiology reports to ensure identification of all potentially eligible patients, as well as consistent and systematic collection of lower respiratory tract samples.

The study has several limitations. It was conducted at a single center, and a significant proportion of patients were already receiving antibiotics at the time of sample collection, potentially affecting microbiological yield. Selection bias may have been introduced by excluding patients who had been treated for NV-HAP with antibiotics for more than 72 hours. Additionally, the absence of a gold standard for pathogen attribution in NV-HAP, coupled with the lack of clinical adjudication to differentiate colonization from true infection, further limits interpretation. The high adherence to national empirical treatment guidelines (86.7%) may also account for the low rate of therapy modification, thereby constraining the evaluation of the added value of multiplex PCR in guiding antibiotic stewardship.

The use of multiplex PCR may offer valuable advantages for clinicians. Nevertheless, it is crucial to acknowledge the limitations of multiplex PCR platforms. They only detect microbes included in the panel [25] and do not differentiate between living and dead organisms. Interestingly, the recently published INHALE study [16] using the same syndromic multiplex PCR platform among intensive care unit (ICU) patients improved antibiotic stewardship, but failed to demonstrate significant differences in all-cause mortality at 28 days, and did not meet the preset noninferiority margin for clinical cure. Our study was not designed to examine the above-mentioned endpoints.

In conclusion, this trial demonstrates that noninvasive sampling from lower respiratory airways is feasible and should be encouraged in NV-HAP. The use of syndromic multiplex PCR provided faster results and a higher detection rate for microbial etiology compared to traditional culture methods. The fact that antimicrobial treatment was not modified more frequently with syndromic multiplex PCR compared to traditional methods may reflect the adequacy of national guidelines for empirical antibiotic therapy and the low prevalence of antimicrobial resistance in our cohort. In sum, we believe syndromic multiplex PCR has a role as a diagnostic adjunct in NV-HAP, but results should be used with the strengths and limitations of the methodology in mind. The trial also supports previous findings on the high morbidity and mortality associated with episodes of NV-HAP.

## Ethics

Signed informed consent was obtained from most patients. In cases where patients had a temporary or permanent inability to provide consent, close relatives or next of kin were consulted to approve or decline participation on the patient's behalf. If no relatives or next of kin were available, study personnel made a temporary inclusion decision based on professional judgment.

The Regional Committee for Medical and Health Research Ethics in Norway approved the study protocol (reference number 78551). The study was also approved by the Data Protection Officer at Vestre Viken Hospital Trust (reference number 20/04324-1). The study was performed according to ethical principles derived from international guidelines, including the Helsinki Declaration and the Council for International Organizations of Medical Sciences, Relevant ICH Good Clinical Practice Guidelines, and relevant laws and regulations, and according to procedures for research in Vestre Viken Hospital Trust.

## Supplementary Material

ofag272_Supplementary_Data

## Supplementary Data

Supplementary materials are available at *Open Forum Infectious Diseases* online. Consisting of data provided by the authors to benefit the reader, the posted materials are not copyedited and are the sole responsibility of the authors, so questions or comments should be addressed to the corresponding author.
