# Supplementary material for: Syndromic Multiplex Polymerase Chain Reaction: The Impact on Microbial Yield in Nonventilator Hospital-Acquired Pneumonia
Source: Open Forum Infect Dis. 2026 May 5;13(5):ofag272. doi: 10.1093/ofid/ofag272 (PMC13195297; doi:10.1093/ofid/ofag272)
Supplement: ofag272_Supplementary_Data [file ofag272_supplementary_data.docx]

SUPPLEMENTARY TABLE 1

| **Viruses** |
| --- |
| Adenovirus |
| Coronavirus |
| Human metapneumovirus |
| Human rhinovirus/enterovirus |
| Influenza A virus |
| Influenza B virus |
| Parainfluenza virus |
| Respiratory syncytial virus |
| Middle East respiratory syndrome coronavirus |
| **Bacteriae** |
| *Acinetobacter calcoaceticus-baumannii* complex |
| *Enterobacter cloacae* complex |
| *Escherichia coli* |
| *Haemophilus influenzae* |
| *Klebsiella aerogenes* |
| *Klebsiella oxytoca* |
| *Klebsiella pneumoniae* group |
| *Moraxella catarrhalis* |
| *Proteus* spp. |
| *Pseudomonas aeruginosa* |
| *Serratia marcescens* |
| *Staphylococcus aureus* |
| *Streptococcus agalactiae* |
| *Streptococcus pneumoniae* |
| *Streptococcus pyogenes* |
| **Atypical bacteria** |
| *Chlamydia pneumoniae* |
| *Legionella pneumophila* |
| *Mycoplasma pneumoniae* |
| **Antimicrobial resistance genes** |
| mecA/mecC and MREJ* |
| KPC** |
| NDM** |
| OXA-48-like*** |
| VIM** |
| IMP** |
| CTX-M** |

Table text: Table detailing the targets of the FilmArray pneumonia plus panel.

*Reported when *S. aureus* is also detected.

** Reported when ACB-complex, EC-complex, *E. coli*, *K. aerogenes*, *K. oxytoca*, *K. pneumoniae* group, *Proteus* spp., *P aeruginosa*, or *S. marcescens* is also detected.

*** Reported when EC-complex, *E. coli*, *K. aerogenes*, *K. oxytoca*, *K. pneumoniae* group, *Proteus* spp., or *S. marcescens* is also detected.

SUPPLEMENTARY TABLE 2

| **Sample ID** | **Bacteria** | **FA (c/mL)** | **Culture** |
| --- | --- | --- | --- |
| 4 | ACB-complex | >10^4 | - |
| 16 | *C. koseri* | - | Plentiful |
| 3 | *E. coli* | >10^6 | - |
| 11 | *E. coli* | >10^6 | Moderate |
| 17 | *E. coli* | >10^4 | - |
| 21 | *E. coli* | >10^4 | - |
| 22 | *E. coli* | >10^4 | - |
| 25 | *E. coli* | >10^4 | - |
| 43 | *E. coli* | >10^5 | - |
| 3 | *E. faecalis* | - | Plentiful |
| 18 | EC-complex | >10^4 | Moderate |
| 22 | EC-complex | >10^7 | - |
| 23 | EC-complex | >10^4 | - |
| 37 | EC-complex | >10^4 | - |
| 41 | EC-complex | >10^6 | - |
| 7 | *H. influenzae* | >10^4 | - |
| 24 | *H. influenzae* | >10^5 | - |
| 25 | *H. influenzae* | >10^6 | - |
| 27 | *H. influenzae* | >10^7 | Plentiful |
| 28 | *H. influenzae* | >10^4 | - |
| 33 | *H. influenzae* | >10^7 | Sparse |
| 36 | *H. influenzae* | >10^6 | - |
| 43 | *H. influenzae* | >10^6 | - |
| 28 | *K. oxytoca* | >10^4 | - |
| 29 | *K. oxytoca* | >10^7 | Plentiful |
| 31 | *K. oxytoca* | >10^5 | Plentiful |
| 39 | *K. oxytoca* | - | Moderate |
| 3 | *K. pneumoniae* | >10^6 | Plentiful |
| 12 | *K. pneumoniae* | >10^5 | Moderate |
| 35 | *K. pneumoniae* | >10^4 | - |
| 5 | *M. catarrhalis* | >10^7 | Plentiful |
| 10 | *M. catarrhalis* | >10^7 | - |
| 14 | *M. catarrhalis* | >10^6 | - |
| 42 | *M. catarrhalis* | >10^6 | - |
| 30 | *P. aeruginosa* | >10^4 | - |
| 2 | *S. agalactiae* | >10^5 | - |
| 30 | *S. agalactiae* | >10^6 | - |
| 4 | *S. aureus* | >10^6 | - |
| 6 | *S. aureus* | >10^6 | Plentiful |
| 8 | *S. aureus* | >10^4 | - |
| 9 | *S. aureus* | >10^4 | - |
| 13 | *S. aureus* | >10^7 | - |
| 15 | *S. aureus* | >10^4 | - |
| 16 | *S. aureus* | >10^6 | Plentiful |
| 17 | *S. aureus* | >10^7 | - |
| 19 | *S. aureus* | >10^7 | - |
| 20 | *S. aureus* | Not ran | Plentiful |
| 26 | *S. aureus* | >10^5 | - |
| 27 | *S. aureus* | >10^6 | - |
| 30 | *S. aureus* | >10^6 | - |
| 33 | *S. aureus* | >10^6 | Sparse |
| 34 | *S. aureus* | >10^5 | - |
| 35 | *S. aureus* | >10^7 | - |
| 37 | *S. aureus* | >10^4 | - |
| 38 | *S. aureus* | >10^7 | Plentiful |
| 44 | *S. aureus* | >10^4 | - |
| 1 | *S. marcescens* | >10^5 | Plentiful |
| 16 | *S. marcescens* | >10^4 | Plentiful |
| 18 | *S. marcescens* | - | Moderate |
| 22 | *S. marcescens* | >10^5 | - |
| 30 | *S. marcescens* | >10^7 | Plentiful |
| 32 | *S. marcescens* | >10^4 | - |
| 24 | *S. pneumoniae* | >10^6 | Moderate |
| 33 | *S. pneumoniae* | >10^7 | Plentiful |
| 40 | *S. pneumoniae* | >10^4 | - |
| 41 | *S. pneumoniae* | >10^7 | Plentiful |
| 42 | *S. pneumoniae* | >10^7 | - |

SUPPLEMENTARY TABLE 3

| **Sample ID** | **Bacteria** | **Benzyl penicillin** | **Gentamicin** | **Cefotaxim** | **Piperacillin/ tazobactam** | **Meropenem** |
| --- | --- | --- | --- | --- | --- | --- |
| 16 | *C. koseri* | R | S | S | S | S |
| 18 | *EC-complex* | R | S | R | R | S |
| 27 | *H. influenzae* | S | ** | S | S | ** |
| 33 | *H. influenzae* | S | ** | S | S | ** |
| 29 | *K. oxytoca* | R | S | S | S | S |
| 31 | *K. oxytoca* | R | S | S | S | S |
| 39 | *K. oxytoca* | R | S | S | S | S |
| 3 | *K. pneumoniae* | R | S | S | S | S |
| 12 | *K. pneumoniae* | R | S | S | S | S |
| 5 | *M. catarrhalis* | R | ** | S | S | ** |
| 6 | *S. aureus* | S | S | I* | ** | ** |
| 16 | *S. aureus* | S | S | I* | ** | ** |
| 20 | *S. aureus* | S | S | I* | ** | ** |
| 33 | *S. aureus* | S | S | I* | S | ** |
| 38 | *S. aureus* | R | S | I* | ** | ** |
| 1 | *S. marcescens* | R | S | S | S | S |
| 16 | *S. marcescens* | R | S | S | S | S |
| 18 | *S. marcescens* | R | S | S | S | S |
| 30 | *S. marcescens* | R | S | S | S | S |
| 24 | *S. pneumoniae* | I | ** | ** | ** | ** |
| 33 | *S. pneumoniae* | I | ** | S | S | ** |
| 41 | *S. pneumoniae* | S | ** | ** | ** | ** |

Table text: Antimicrobial resistance to antibiotics in empirical regiments.

*Inferred from cefoxitin screening

**Susceptibility testing not performed

SUPPLEMENTARY TABLE 4

| **Agreement and Cohen's kappa** | | | | |
| --- | --- | --- | --- | --- |
| **Bacteria** | **Agreement** | **Kappa ± SD** | **Number of detections** | |
|  |  |  | **Culture** | **mPCR** |
| ACB-Complex | Not calculable | Not calculable | 0 | 1 |
| *Enterobacter cloacae* complex | 94.2 % | 0.2612±0.0967 | 2 | 5 |
| *E. Coli* | 93.0 % | 0.2344±0.0694 | 1 | 7 |
| *H. Influenzae* | 93.0 % | 0.3768±0.0843 | 2 | 8 |
| *K. aerogenes* | Not calculable | Not calculable | 0 | 0 |
| *K. oxytoca* | 97.7 % | 0.6546±0.1078 | 3 | 3 |
| *K. pneumonia* group | 98.8 % | 0.7943±0.1055 | 2 | 3 |
| *M. Catarrhalis* | 96.5 % | 0.3886±0.0853 | 1 | 4 |
| *Proteus* spp. | Not calculable | Not calculable | 0 | 0 |
| *P. aeruginosa* | Not calculable | Not calculable | 0 | 1 |
| *S. Aureus* | 82.6 % | 0.3288±0.0877 | 5 | 19 |
| *S. Marcescens* | 96.5 % | 0.6485±0.1071 | 4 | 5 |
| *S. Agalactiae* | Not calculable | Not calculable | 0 | 2 |
| *S. Pneumoniae* | 97.7 % | 0.7386±0.1041 | 3 | 5 |
| *S. Pyogenes* | Not calculable | Not calculable | 0 | 0 |
| **Overall agreement, any detection** | **69.1 %** | **0.3889±0.0901** | **23** | **63** |

Table text: Agreement and Cohen’s kappa for individual bacteria and for overall agreement. Kappa was not only calculable for bacteria in the mPCR panel, and only for bacteria where both mPCR and culture had detections.
